# MYC at the tumor–immune interface: mechanisms of immune escape and immunotherapy resistance

**DOI:** 10.3389/fimmu.2026.1738440

**Published:** 2026-01-23

**Authors:** Íñigo González-Larreategui, Manrique Valdés-Bango Martín, Sílvia Casacuberta-Serra, Laura Soucek

**Affiliations:** 1 Models of Cancer Therapies Laboratory, Vall d’Hebron Institute of Oncology, Cellex Centre, Hospital University Vall d’Hebron Campus, Barcelona, Spain; 2 Peptomyc S.L., Barcelona, Spain; 3 Institució Catalana de Recerca i Estudis Avançats, Barcelona, Spain; 4 Department of Biochemistry and Molecular Biology, Universitat Autonoma de Barcelona, Bellaterra, Spain

**Keywords:** immune evasion, MYC, resistance, targeted therapies, tumor microenvironment

## Abstract

Immunotherapies have transformed cancer treatment by harnessing the immune system to recognize and eliminate malignant cells, offering durable clinical benefit across diverse tumor types. Despite successes with immune checkpoint inhibitors (ICIs) and other strategies like cytokines, oncolytic viruses, cancer vaccines, bispecific antibodies, and adoptive cell therapies, substantial fractions of patients still fail to respond or develop resistance. The oncogene MYC, deregulated in ~70% of human cancers, has emerged as a central driver of immune evasion and a key contributor to immunotherapy failure. MYC regulates broad transcriptional networks controlling proliferation, metabolism, angiogenesis, and cell survival, while also orchestrating profound remodeling of the tumor microenvironment (TME). Mechanistically, oncogenic MYC suppresses antigen processing and presentation, attenuates interferon signaling, and upregulates immune checkpoints such as PD-L1 and CD47. Concurrently, MYC stimulates secretion of immunosuppressive cytokines and chemokines that recruit regulatory T cells, myeloid-derived suppressor cells, and M2 macrophages, while driving metabolic reprogramming that fosters nutrient competition, hypoxia, and acidosis, impairing effector T- and NK-cell function. Through these pathways, MYC promotes primary, adaptive, and acquired resistance to immunotherapy. Targeting MYC, directly or indirectly, holds promise to restore immune surveillance and potentiate immunotherapeutic efficacy. This review highlights MYC as a master regulator of tumor–immune interactions and underscores the therapeutic potential of MYC inhibition to overcome resistance and expand the clinical impact of cancer immunotherapy.

## Immunotherapies in cancer

Normally, our immune system is quite effective in controlling diseases, but it often fails to keep cancer at bay. This paradox was first studied in the late 19th century (1–3), but the concept of immunosurveillance only emerged by the mid-20th century. According to it, immune cells and their secreted molecules can distinguish between self-tissues and foreign threats (4), opening the opportunity to harness the immune system against malignancies like cancer. In this context, the discovery of various cells from both the innate and adaptive immune responses, along with the cytokines involved, has shaped the field of immunotherapy. Cancer immunotherapy represents a revolutionary shift in cancer care, complementing and sometimes replacing chemotherapy in various tumor types, where it offers clinical benefits with a usually more favorable toxicity profile (5). Today, immunotherapy treatments are available for more than 30 types of solid tumors and hematologic malignancies, often inducing durable responses in patients with advanced or otherwise difficult-to-treat cancers (6).

### Cytokine-based therapies

The earliest immunotherapies developed were cytokine-based. Cytokines serve as “messengers” orchestrating cellular interactions within the immune system in response to stresses. Interferon type 1 (IFN-I) and interleukin-2 (IL-2) were the first cytokines to be identified and FDA-approved by the end of the 20th century (7–10). Several additional cytokines followed the same path but displayed toxic side effects that prompted researchers to explore other immune components for cancer therapy.

### Oncolytic viruses

Another category of agents is based on oncolytic viruses, which take advantage of the weakened antiviral defenses of cancer cells. Genetic engineering has led to the approval of four oncolytic virus therapies worldwide, used against melanoma, head and neck cancers, and malignant glioma (11–14).

### Cancer vaccines

Cancer vaccines using tumor-specific antigens to elicit T-cell-mediated anti-tumor immune responses emerged as a less toxic alternative than cytokines. This concept initially arose from the use of Bacillus Calmette-Guérin (BCG), a weakened form of *Mycobacterium bovis*, as a vaccine to reduce tumor growth (15–17). However, it was not until 2010 that the FDA approved sipuleucel-T, the first cancer vaccine (18). Advances in cancer immunotherapy have led to next-generation vaccines using mRNA technology and tumor neoantigens.

### Antibody-based therapies

With the identification of tumor antigens capable of mediating T-cell responses, antibody-based therapies emerged as a new possible approach. These antibodies either bind tumor cell antigens to mark them for immune destruction or target immune cells to activate them against cancer cells. Antibodies targeting immune cell receptors began with the discovery of 4-1BB, a T-cell co-stimulatory receptor (19–21). This discovery paved the way for monoclonal antibodies developed to amplify T-cell responses. On the other hand, the identification of immune checkpoints (IC) revolutionized immunotherapy with immune checkpoint inhibitors (ICIs) designed to block inhibitory receptors on immune cells and reactivate anti-tumor immunity (3). CTLA-4, PD-1 receptor and its ligand, PD-L1, are the most common checkpoint molecules therapeutically targeted by approved therapies for various cancers (22–32). After them, other T cell surface receptors, such as LAG-3 and TIM-3, also became target for ICIs (1). To date, 128 ICIs have already been approved, representing 81% of all cancer immunotherapies (6). Bispecific antibodies are the next generation of antibody-based therapies combining checkpoint blockade with targeted co-stimulation of T cells. So far, 8 bispecific antibodies have received FDA approval and are emerging as important immunotherapies (6).

### Adoptive cell therapies

More recently, the power of T cells to make potent cancer-targeting therapies led to the development of adoptive cell therapies. They utilize patients’ or donors’ immune cells, which are collected, sometimes genetically engineered, expanded, and reinfused to treat cancer. These personalized treatments began with the use of tumor-infiltrating lymphocytes (TILs) (33). However, the identification of T-cell receptor (TCR) genetic sequences enabled the development of TCR-engineered T cells and chimeric antigen receptor (CAR) T cells (34, 35). The challenge of short-lived responses was addressed by the addition of co-stimulatory domains like CD28 or 4-1BB, which enhanced durability and potency (36–38), leading to several FDA approvals for hematological malignancies (39–41) and solid tumors (42). Importantly, CAR technology has lately expanded beyond T cells to natural killer (NK) cells and macrophages, offering off-the-shelf products accessible to more patients (43).

However, despite notable successes, immunotherapies still fail to benefit a large number of patients, mainly due to tumor-intrinsic heterogeneity and tumor-extrinsic factors like the immunosuppressive tumor microenvironment (TME). Hence, unlocking immunotherapy’s full potential requires strategies to overcome such resistance mechanisms. Resistance can be broadly categorized as primary, adaptive, or acquired, each shaped by distinct intrinsic and extrinsic processes that undermine antitumor immune responses (44). Primary resistance describes the absence of clinical benefit from the onset of treatment, occurring when tumors fail to trigger an effective immune response despite therapy (45). In contrast, adaptive resistance emerges under therapeutic pressure, as tumor cells dynamically reprogram immune-recognition pathways to evade immune destruction. This process reflects the complex crosstalk between tumor and immune cells within the TME, as well as the connection between intrinsic and extrinsic factors (44, 46). Finally, acquired resistance denotes the loss of therapeutic benefit typically due to epigenetic remodeling, epithelial–mesenchymal transition (EMT), stemness induction, and mitochondrial or caspase alterations. Altogether, these resistance mechanisms highlight the diverse ways tumors escape immunotherapy, and they encompass both tumor-intrinsic drivers and microenvironmental influences.

Among these drivers, the MYC oncogene has emerged as a key orchestrator of immune evasion and immunotherapy resistance. Through its key role in tumor proliferation, metabolism, and TME remodeling, MYC shapes both intrinsic tumor properties and the extrinsic immune landscape. Moreover, MYC deregulation emerges as an oncogenic feature across the different tumor types with approved immunotherapies (**Table 1**), including melanoma (47–49), renal cell carcinoma (50), esophageal cancer (51), glioblastoma (52), head and neck squamous cell carcinoma (53), prostate cancer (54), breast cancer (55, 56), endometrial cancer (56), non-small-cell lung cancer (57, 58), small-cell lung cancer (59), hepatocellular carcinoma (60), colorectal cancer (57, 61, 62), sarcoma (63–65) and hematological malignancies (66–69). This shared MYC dependency strengthens its potential as a therapeutic target to overcome immunotherapy resistance. Therefore, this review discusses MYC’s role in tumorigenesis, with a particular focus on how MYC rewires antitumor immunity and contributes to immunotherapy failure and highlights therapeutic avenues to target MYC-driven resistance.

**Table 1:** MYC involvement in major tumor types with approved immunotherapies.

Immunotherapy category	Cancer type	MYC involvement
Cytokine-based therapies	Hairy cell leukemia	BRAF as oncogenic driver upstream of MYC (49, 70, 71)
	Melanoma	MYC overexpression is found in 34% of melanomas and associated with treatment resistance (47, 48). Moreover, MYC is downstream of BRAF signaling (49)
	Renal cell carcinoma (RCC)	MYC is overexpressed in 20% of human RCC (50)
Oncolytic viruses	Esophageal cancer	MYC amplification worsen ICI therapy response via PD-L1 in esophageal squamous cell carcinoma (51)
	Glioblastoma (GBM)	MYC activity is associated with initiation and progression of GBM (52)
	Head and neck squamous cell carcinoma (HNSCC)	MYC is rarely mutated in HNSCC (1.2%) but amplified in around 12% of cases (53)
	Melanoma	(47–49)
Cancer vaccines	Prostate cancer	MYC overexpression is a major driver of prostate cancer (54)
Antibody-based therapies	Hematological malignancies	MYC activation by translocation is found in many types of lymphomas and multiple myeloma (66–69)
	Melanoma	(47–49)
	Breast cancer	MYC amplification is observed in approximately 25% of patients (56), and disproportionally elevated in TNBC (55)
	Endometrial cancer	MYC is amplified in almost 11% of endometrial cancers (56)
	Non-small-cell lung cancer (NSCLC)	MYC amplification is observed in 31% of patients (58). Moreover, MYC is stabilized through many mechanisms in KRAS-driven NSCLC tumors (57)
	Small-cell-lung cancer (SCLC)	L-MYC is particularly overexpressed in SCLC (59)
	Hepatocellular carcinoma (HCC)	MYC overexpression and activation is a common event in the pathogenesis of HCC (60)
	Colorectal cancer (CRC)	MYC is downstream of KRAS pathway, which is the oncogenic driver in 50% of CRC (57). In addition, MYC is associated to stress adaptation in KRAS-driven CRC (61, 62)
Adoptive cell therapies	Hematological malignancies	(66–69)
	Melanoma	(47–49)
	Sarcoma	MYC overexpression or deregulation is involved in the tumorigenesis of different sarcoma types (63–65)

## MYC role in cancer

**Figure 1:**
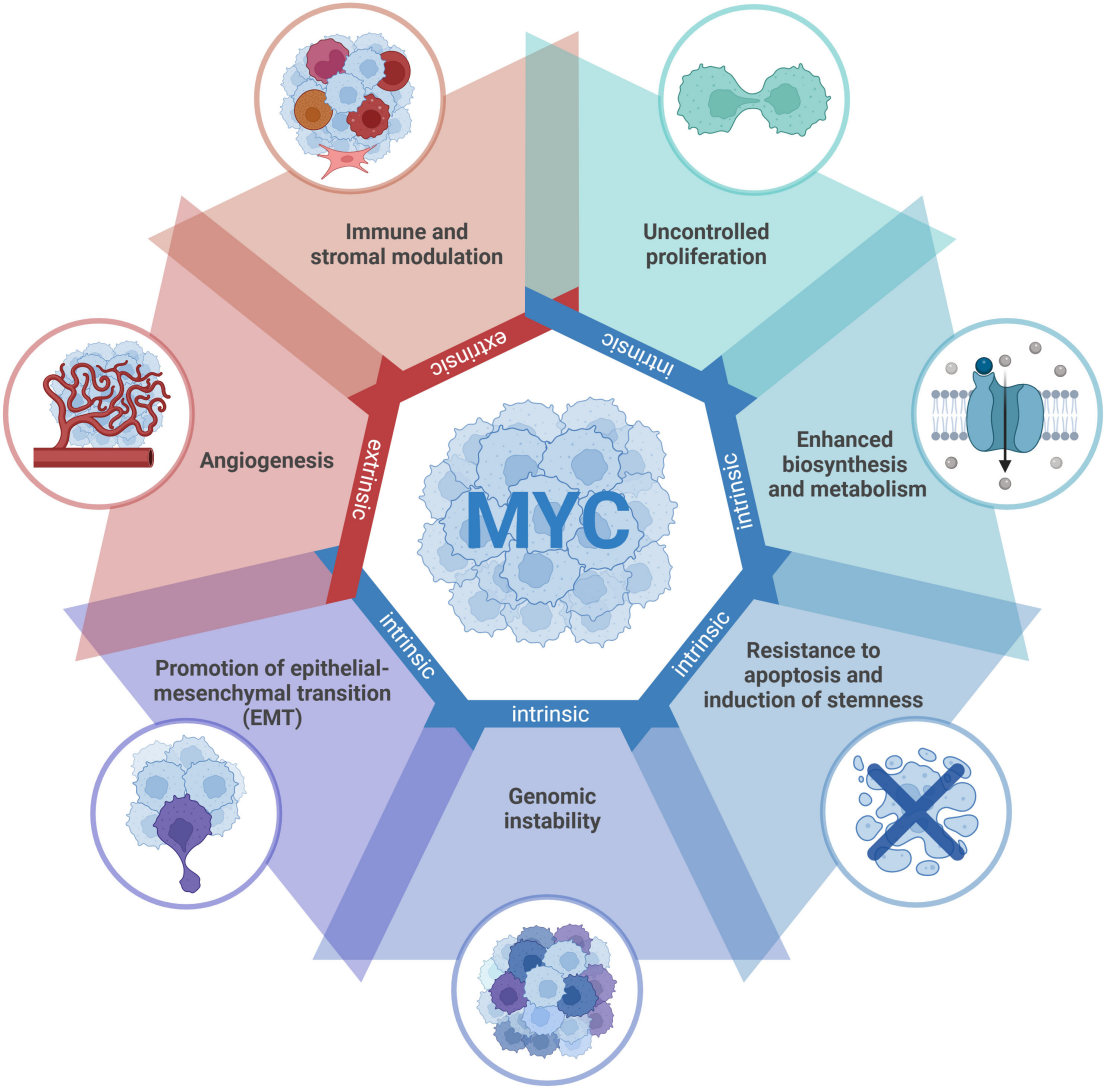
MYC orchestrates diverse tumorigenic programs. Deregulated MYC in cancer cells drives a broad range of cell-intrinsic processes, including uncontrolled proliferation, enhanced biosynthesis and metabolism, resistance to apoptosis and induction of stemness, genomic instability, and epithelial–mesenchymal transition (EMT). At the cell-extrinsic level, MYC profoundly remodels the tumor microenvironment by promoting angiogenesis and modulating immune and stromal components.

MYC is a pleiotropic transcription factor critically involved in tumor initiation, progression, and maintenance (**Figure 1**) (72). Since its discovery over four decades ago, MYC has emerged as one of the most frequently deregulated oncogenes across human malignancies (approximately 70% of all human cancers) (72).

The MYC family comprises three paralogs: c-MYC, N-MYC (MYCN), and L-MYC (MYCL), each with distinct expression patterns and tissue specificities. c-MYC exhibits the broadest expression profile across tissue types and is the most frequently dysregulated member in both hematological and solid tumors (66, 73). N-MYC is predominantly expressed in neural tissues and early hematopoietic development, with its amplification serving as a hallmark of aggressive neuroblastoma (74, 75). L-MYC, on the other hand, shows more restricted expression, primarily in lung tissue, and is particularly overexpressed in small cell lung cancer (SCLC) (59). Across cancer types, MYC deregulation consistently correlates with aggressive disease, therapeutic resistance, and poor clinical outcomes, establishing it as a critical determinant of tumor behavior and treatment response (66, 76).

Under physiological conditions, MYC expression is subject to a very tight temporal and spatial regulation. MYC proteins are virtually absent in quiescent cells but rapidly induced upon mitogenic stimulation, with both mRNA and protein exhibiting short half-lives of 15–20 minutes (72, 77, 78). This rapid turnover ensures precise, dynamic control of MYC-dependent programs through multilayered regulation at transcriptional, post-transcriptional, and post-translational levels (79). At the protein level, MYC stability is governed by sequential phosphorylation events: Serine 62 (S62) phosphorylation promotes stability, while subsequent Threonine 58 (T58) phosphorylation marks MYC for ubiquitin-mediated proteasomal degradation (80). Functionally, MYC operates as an intrinsically disordered protein that requires heterodimerization with its obligate partner MAX through their respective b-HLH-LZ domains to achieve structural competence for DNA binding (81, 82). Then, the MYC-MAX heterodimer recognizes canonical E-box motifs (CACGTG) in target gene regulatory regions, recruiting transcriptional cofactors via MYC N-terminal transactivation domain (TAD) to regulate approximately 15% of the genome (83–85). Beyond activation, MYC also functions as a transcriptional repressor through protein-protein interactions, notably with MIZ-1, which mediates repression of cell cycle inhibitors and anti-apoptotic genes like BCL-2 (86–89). This dual capacity for activation and repression under normal circumstances enables MYC to coordinate proliferation with apoptotic sensitization, preventing uncontrolled growth.

In contrast to many oncogenes and tumor suppressors, the protein-coding sequence of MYC is rarely mutated in cancer. Unlike most cancer-causing genes that are typically altered through a single mechanism, MYC is uniquely subject to deregulation through many diverse processes. These mechanisms include gene amplification (accounting for 15-20% of cases), chromosomal translocations (particularly in leukemias and lymphomas), enhancer hijacking and super-enhancer activation, viral insertion, increased oncogenic signaling (e.g. KRAS, WNT-β-catenin, SRC, RTKs, and NOTCH) that elevates MYC expression and/or protein stability, and post-translational mechanisms including loss of E3 ubiquitin ligases that normally target MYC for degradation (80, 90). Even inactivation of tumor suppressor genes, particularly of APC, PTEN, and PP2A, contributes to MYC stabilization and accumulation (91, 92).

This mechanistic diversity underscores MYC’s central position in oncogenic networks and its capacity to integrate multiple upstream oncogenic signals. Critically, even modest increases in MYC expression, as little as 2-fold above physiological levels, are sufficient to drive tumorigenic processes (93). In such contexts, MYC not only amplifies established transcriptional programs through canonical E-box binding but also engages lower-affinity non-canonical sites in a dose-dependent manner, activating previously silent oncogenic programs (94, 95). Thus, even when it is not the primary driver mutation, MYC functions as a central integration node for both extracellular and intracellular oncogenic signals.

Building on this molecular framework, MYC drives many, if not all, of the hallmarks of cancer by orchestrating a broad array of cell-intrinsic (autonomous) and microenvironmental (extrinsic) tumorigenic programs. The tumorigenic programs driven by MYC have been described in detail elsewhere (57, 96–100) and are summarized schematically in **Figure 1**. Briefly:

- At the cell-intrinsic level, deregulated MYC promotes a variety of tumor cell–autonomous processes, including a) uncontrolled proliferation: MYC promotes cell cycle entry and progression through repression of key inhibitors such as *p16* and *p21*, allowing escape from senescence and cell cycle arrest even under genotoxic stress (101, 102); b) enhanced biosynthesis and metabolism: MYC stimulates ribosomal biogenesis and globally rewires metabolic pathways, upregulating nutrient transporters and glycolytic/glutaminolytic enzymes to sustain rapid biomass accumulation (103, 104); c) resistance to apoptosis and induction of stemness: MYC enhances DNA replication, suppresses apoptotic programs, and induces stem-like transcriptional states associated with tumorigenesis and therapeutic resistance (105–107); d) genomic instability: chronic MYC activation induces DNA breaks, chromosomal aberrations, and reactive oxygen species (ROS) production, fueling tumor evolution and heterogeneity (106, 108, 109); e) promotion of epithelial–mesenchymal transition (EMT): MYC directly represses E-cadherin and induces SNAIL and other EMT regulators, promoting invasion and metastasis (110, 111).
- At the cell-extrinsic level, MYC profoundly remodels the tumor microenvironment to sustain tumor growth through a) angiogenesis: MYC represses anti-angiogenic signaling and factors, including thrombospondin-1 (TSP1)-dependent angiogenic switch, to promote vascularization and nutrient supply (112–114); b) immune and stromal modulation: MYC reprograms immune and stromal components of the TME to create conditions conducive to tumor persistence and dissemination, which ultimately contributes to resistance to IO therapies - mechanisms that will be discussed in detail below (57, 76, 90, 115, 116).

### MYC role in immune evasion

**Figure 2:**
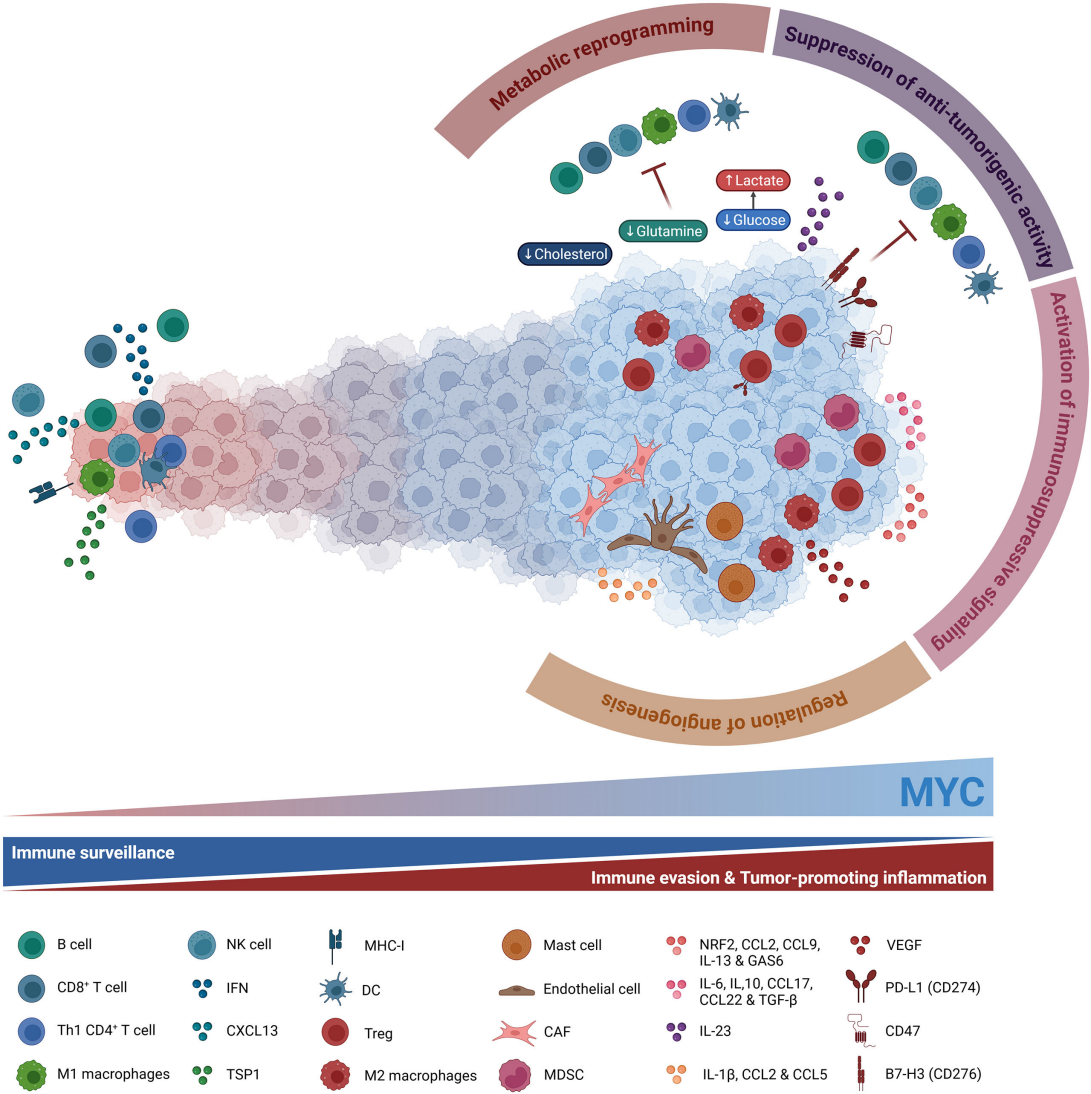
MYC blocks immune surveillance and drives immune evasion and tumor-promoting inflammation. Deregulated MYC in cancer cells modulates cytokine signaling, upregulates molecules that facilitate immune escape, and secretes factors that remodel the tumor microenvironment (TME). MYC activates immunosuppressive signaling pathways through the secretion of inhibitory cytokines, recruiting immunosuppressive and tumor-promoting populations such as myeloid-derived suppressor cells (MDSC), regulatory T cells (Treg) (via IL-6, IL-10, CCL17, CCL22 and TGF-β secretion), and immunosuppressive M2 macrophages (via NRF2, CCL2, CCL9, IL-13 and GAS6 secretion). MYC also suppresses anti-tumorigenic activity by downregulating MHC-I, IFN and IFN-derived molecules such as CXCL13 and by increasing IL-23 secretion, diminishing dendritic cell (DC) function and restricting the infiltration of CD8^+^ T cells, Th1 CD4^+^ T cells, B cells, and NK cells. Moreover, MYC-dependent upregulation of immune checkpoint molecules (PD-L1, CD47, B7-H3) in cancer cells further inhibits T and NK cell-mediated surveillance. Additionally, MYC creates an immunosuppressive niche through metabolic reprogramming, by consuming glucose, glutamine and cholesterol, acidifying the TME by lactate secretion. MYC is also capable of enhancing angiogenesis by directly repressing thrombospondin-1 (TSP1) or indirectly by secreting IL-1β, CCL2 and CCL5, recruiting endothelial cells, mast cells and cancer-associated fibroblasts (CAF), along with VEGF secretion by M2 macrophages. All these MYC-driven activities create an immune-privileged niche for immune evasion and cancer progression.

MYC is a central orchestrator of tumor immune evasion and a key mediator of resistance to IO therapies (57, 90, 115, 116). In fact, MYC modulates cytokine signaling, upregulates molecules that help cancer cells evade immune surveillance and secret compounds that recruit immunosuppressive and tumor-promoting cells (**Figure 2**).

#### Activation of immunosuppressive signaling

More in detail, the presence of deregulated MYC in cancer cells can activate immunosuppressive signaling pathways through the secretion of inhibitory cytokines that ultimately facilitate immune evasion (76, 90, 117). It stimulates the secretion of interleukins IL-6, IL-10, cytokines CCL17, CCL22, and transforming growth factor beta (TGF-β), attracting myeloid-derived suppressor cells (MDSCs) and regulatory T cells (Tregs) to the tumor site (118–121). MYC-deregulated tumors can also produce NRF2, CCL2, CCL9, IL-13, and GAS6, recruiting macrophages and polarizing them into their immunosuppressive state, known as M2 state (118, 122–126). Additionally, MYC-driven tumors release the immunosuppressive molecules IL-1β, CCL2, and CCL5, promoting the recruitment of endothelial and mast cells that, together with vascular endothelial growth factor (VEGF) secretion by M2 macrophages, enhance angiogenesis (118, 120, 127).

#### Suppression of anti-tumorigenic activity

Beyond activating immunosuppressive pathways, MYC-deregulated tumors also suppress anti-tumorigenic activity, both by inhibiting anti-cancer signaling and by activating immune checkpoint molecules. One of the primary mechanisms repressed by MYC is antigen processing and presentation. In this context, MYC downregulates both MHC-I and MHC-II molecules in several cancers, reducing the stimulation of cytotoxic T cells and NK cells, while diminishing the presentation activity of helper T cells (118, 128–133). Another signaling mechanism silenced in the presence of oncogenic MYC is the interferon pathway. In models of neuroblastoma, triple-negative breast cancer (TNBC) and pancreatic ductal adenocarcinoma (PDAC), MYC attenuates the expression of type I interferons and IFN-derived molecules such as STAT1 and STAT2, lessening T cell response. This attenuation of the IFN pathway further limits CXCL13 production and reduces NK cell and B cell tumor infiltration (132, 134, 135). In addition, MYC-induced IL-23 secretion functions as a critical immunomodulatory factor that actively prevents T cells, B cells, and NK cells from infiltrating the tumor microenvironment (118, 125, 136). Moreover, MYC-deregulated tumors frequently upregulate immune checkpoint molecules to effectively neutralize anti-tumor immune responses and establish a protective immunosuppressive barrier. Three of these molecules are PD-L1 (CD274), CD47, and B7-H3 (CD276). Numerous studies have established the correlation between hyperactivated MYC and overexpression of the first two immune checkpoints: deregulated MYC directly induces the expression of both PD-L1 and CD47, interfering with the crosstalk between tumor cells and dendritic cells, weakening T cell response, and ultimately enhancing immune escape (51, 117, 118, 137–141). In the case of B7-H3 immune checkpoint, its MYC-driven overexpression inhibits T and NK cell-mediated immune surveillance (142, 143). All these MYC-derived modulations effectively create an immune-privileged niche for cancer progression.

#### Metabolic reprograming

As summarized in **Figure 2**, MYC also plays a crucial role in immunosuppression through metabolic reprogramming. Deregulated MYC boosts both glucose and glutamine metabolism in cancer cells, leading to T and NK cell suppression via nutrient competition, reduction of antigen presentation and fostering an immunosuppressive microenvironment by attracting M2 macrophages, Tregs, and MDSCs (90, 98, 115). MYC-amplified tumors orchestrate a fundamental metabolic shift from oxidative phosphorylation to glycolysis, known as Warburg effect, through the coordinated overexpression of key glycolytic enzymes including GLUT1, HK2, and LDHA (144–147). This leads to lactate accumulation, acidifying the tumor microenvironment and shifting it towards an immunosuppressive phenotype through attenuation of dendritic cells, M2 polarization of macrophages, suppression of cytolytic activity of T and NK cells, and overexpression of PD-1 by Tregs (148–152). In the case of glutamine metabolism, MYC leads to overexpression of glutamine synthetase (GS) (153), and extreme glutamine consumption by tumor cells creates a severely glutamine deprived environment, forcing T cells into a dysfunctional state with reduced cytotoxic activity without affecting Tregs, which can survive in glutamine-poor conditions (154, 155). Additionally, MYC upregulates glutaminase (GLS), modulating signaling pathways that promote the recruitment of M2 macrophages, Tregs, and MDSCs (156, 157). Lipid metabolism modulation by MYC has also been identified as a process vital for tumor cell survival and proliferation (158). MYC activity in some NSCLC and CRC enhances cholesterol intake and storage, which in turn reduces MYC ubiquitination and degradation while promoting all the MYC-associated immunosuppressive pathways (158–160). Related to this, MYC promotes *de novo* fatty acid synthesis and broader lipidogenesis by transcriptionally activating key lipogenic enzymes and cooperating with SREBP1 and other metabolic regulators. This supports membrane biogenesis, signaling lipids and energy storage in MYC-driven cancers (161).

#### Regulation of angiogenesis

Closely linked to the metabolic reprogramming of cancer cells to efficiently proliferate and adapt to reduced oxygen and nutrient availability is MYC capacity to enhance angiogenesis. As already mentioned before and illustrated in **Figure 2**, deregulated MYC can directly repress the expression of anti-angiogenic factors like TSP1 (112–114, 162, 163), or indirectly enhance angiogenesis via immunosuppressive cells. MYC-driven recruitment of endothelial and mast cells, along with VEGF secretion by M2 macrophages, stimulates angiogenesis in MYC-driven tumors (118, 120, 127). These myeloid-derived cells can also overexpress PD-L1, which further enhances the process (118). Furthermore, MYC-driven angiogenesis can also be induced by immune cells such as cancer-associated fibroblasts (CAFs). MYC-driven CAFs secrete cytokines that promote angiogenesis and lymphangiogenesis (112). These characteristics complement the already established function of CAFs in promoting cell physical exclusion, as well as altering glucose metabolism by upregulating glycolytic enzymes, again reinforcing the intimate connection between metabolic reprogramming and angiogenesis in creating a tumor-promoting microenvironment (76, 164, 165).

While this review focuses on MYC as a central regulator of tumor immune evasion, immune responses are ultimately shaped by a complex interplay of oncogenic and tumor-suppressor pathways. Alterations in genes such as TP53, mismatch repair deficiency (dMMR), oncogenic KRAS, and tumor suppressors such as STK11/LKB1 are well-established modulators of tumor immunogenicity, influencing antigen presentation, interferon signaling, metabolic programs, and response to immune checkpoint blockade. Importantly, mutations in these pathways, and their co-occurrence, can substantially influence how MYC operates within both tumor cells and the tumor immune microenvironment, and MYC activity can, in turn, modulate the immune consequences of these alterations. As a result, the impact of MYC on tumor-intrinsic programs and immune regulation is highly tumor-specific and tissue-dependent. In this context, MYC functions less as an isolated driver and more as a transcriptional integrator that coordinates immune-relevant programs in a manner shaped by genetic and tissue context. Comprehensive discussions of these additional pathways have been reviewed extensively elsewhere (166–173).

### MYC as a mechanism of resistance to immunotherapy

**Figure 3:**
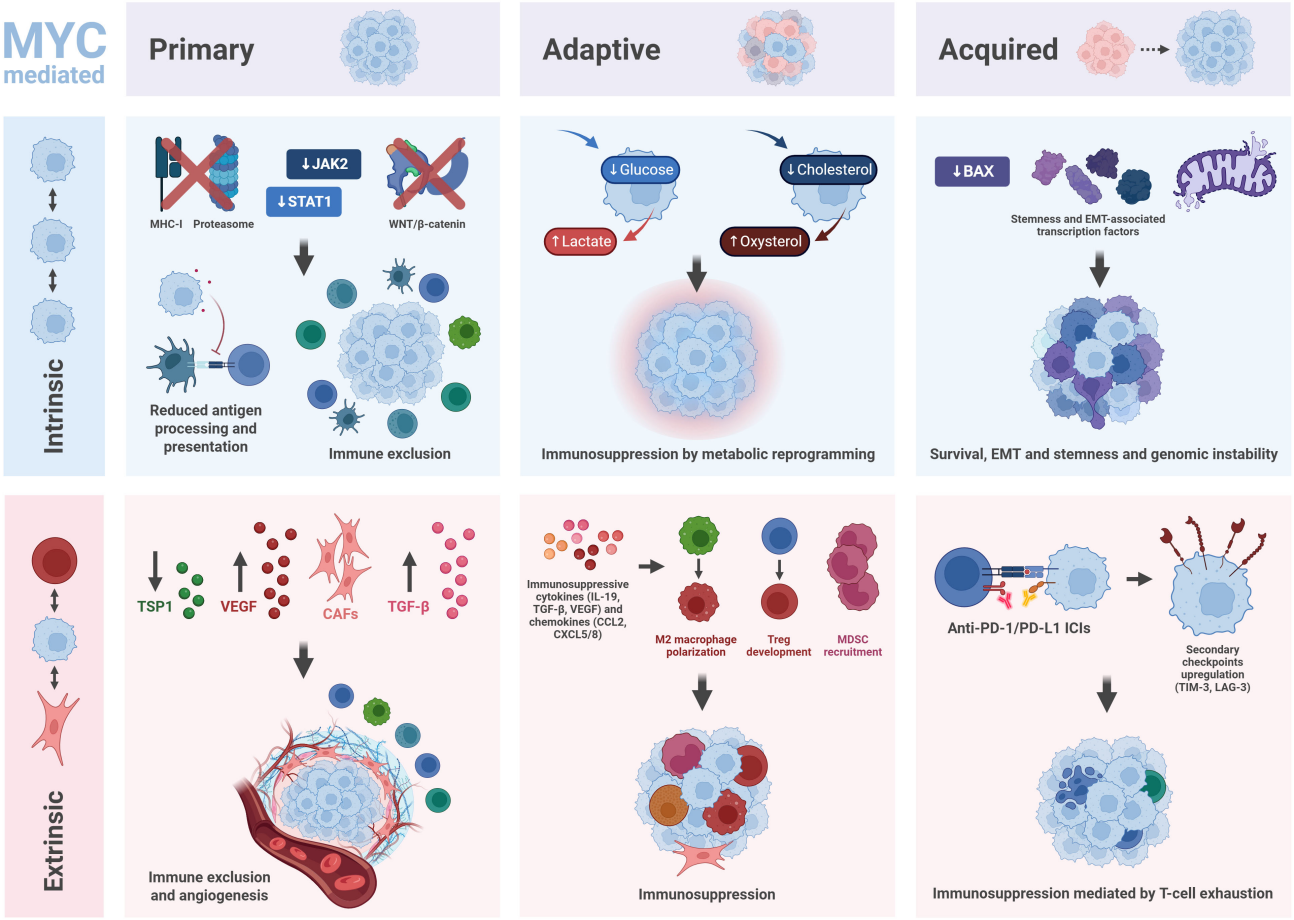
MYC mediates several mechanisms of resistance to immunotherapy. MYC influences intrinsic and extrinsic processes in primary, adaptive and acquired resistance mechanisms. In primary resistance, deregulated MYC represses MHC-I and proteasome expression, IFN-derived JAK2 and STAT1, and WNT/β-catenin signaling, reducing antigen processing and presentation and contributing to immune exclusion. At the extrinsic level, MYC-derived VEGF and TGF-β activation, thrombospondin-1 (TSP1) repression and cancer associated fibroblasts (CAF) recruitment result in immune exclusion and enhanced angiogenesis. MYC regulates adaptive resistance by converting glucose and cholesterol into lactate and oxysterol, reprogramming tumor metabolism and promoting immunosuppression. Extrinsically, deregulated MYC fosters immunosuppressive cytokine (IL-19, TGF-β and VEGF) and chemokine (CCL2, CXCL5 and CXCL8) secretion, promoting M2 macrophage polarization, regulatory T cell (Treg) development and myeloid-derived suppressor cell (MDSC) recruitment, creating an immunosuppressive niche. In acquired resistance, MYC represses pro-apoptotic mediators such as BAX, induces stemness and EMT-associated transcription factors and disrupts mitochondrial structure, influencing apoptotic sensitivity and genomic stability. At the extrinsic level, MYC impairs anti-PD-(L)1 efficacy by upregulating secondary checkpoints such as TIM-3 and LAG-3, mediating T-cell exhaustion. Thus, MYC plays a pivotal role in the generation of resistance to immunotherapy.

MYC deregulation rewires tumor-intrinsic signaling pathways to escape immune surveillance, while also remodels the TME to suppress antitumor immunity and reduce immunotherapy efficacy. It directly influences intrinsic and extrinsic mechanisms in primary, adaptive or acquired resistance to immunotherapy (**Figure 3**).

#### Primary resistance

In the case of primary resistance, tumor-intrinsic factors such as the lack of neoantigen expression represent key drivers of ineffective immune responses from the onset of treatment. Loss or alteration of tumor-specific antigens—through antigen shedding, epitope mutations from genetic instability, or endocytic-antigen formation—facilitates immune evasion (174–176). Additionally, defects in antigen processing or presentation, such as impaired proteasome function or genetic and epigenetic alterations of MHC can contribute to immunotherapy resistance (177). Other intrinsic factors related to resistance include loss or mutation of beta 2 microglobulin (B2M), which disrupts the MHC-I complex, impairing antigen display (178). MYC overexpression attenuates antigen processing and presentation by repressing MHC-I and proteasome gene expression, thereby mimicking antigen-loss phenomena such as B2M deletion (179). These resistance mechanisms have been linked to MYC in NSCLC and CRC cancer models. Aberrant signaling pathways, particularly those linked to interferon-gamma (IFNγ), further promote resistance by upregulating PD-L1 or recruiting immunosuppressive lymphocytes (180). Prolonged IFNγ activation or mutations in the JAK1/2-STAT1 axis impair antigen presentation and favor tumor immune evasion (181, 182). Deregulated MYC can disrupt these pathways, including IFNγ signaling, through transcriptional suppression of JAK2 and STAT1, which limits effector T-cell recruitment and promotes immune exclusion in SCLC cell lines (183, 184). Additionally, dysregulation of other signaling pathways such as MAPK, PI3K and WNT pathways suppresses immune infiltration and activation. MAPK activation increases VEGF and IL-8, limiting cytotoxic lymphocytes, while WNT/β-catenin signaling excludes dendritic cells from tumors (185, 186). The WNT/β-catenin pathway is likewise influenced by MYC, contributing to dendritic cell exclusion within the TME (187). Together, these alterations hinder antigen processing, presentation and effector immune cell recruitment, collectively reducing immunotherapy efficacy and illustrating the complex molecular landscape underlying tumor immune escape mechanisms.

Tumor-extrinsic influences within the TME—such as stromal barriers, aberrant vasculature, and TGF-β-driven immunosuppression—further reinforce resistance. Dense stroma, made of extracellular matrix and fibroblasts, physically blocks T cells from entering tumors. Targeting stromal components like FAP of FAK can improve T-cell penetration and sensitivity to immunotherapy (188–190). Aberrant vasculature also worsens immune exclusion. The abnormal, disorganized tumor blood vessels impair efficient immune cell trafficking. Endothelial cells lining these vessels often express suppressive molecules that block T-cell adhesion and extravasation. In this context, vascular normalization therapy aims to restore healthy vessel function, improving immune cell access and treatment response (191–193). As depicted in **Figure 3**, MYC deregulation in cancer cells contributes to these extrinsic mechanisms by promoting angiogenesis and fibroblast-mediated fibrosis, which reshapes the TME into an immune-excluding niche. As already mentioned in previous sections, deregulated MYC induces VEGF, angiopoietin-2, and IL-8, while repressing anti-angiogenic factor TSP1, resulting in disorganized, leaky vasculature that fosters hypoxia and limits immune infiltration (112). Complementary to this, TGF-β secretion by both tumor and stromal cells can also potentiate immunosuppression, by inducing Tregs, inhibiting effector T-cell function, and promoting stromal fibrosis and abnormal vasculature. Moreover, high TGF-β signaling correlates with poor immune infiltration and resistance to PD-1/PD-L1 blockade (194). In this context, MYC overexpression activates TGF-β, PDGF and IL-6 signaling, stimulating fibroblast differentiation into CAFs that deposit collagen and fibronectin, which ultimately stiffens the matrix and blocks cytotoxic cells in different breast cancer subtypes (195, 196).

#### Adaptive resistance

The TME is profoundly influenced by the high metabolic demands of cancer cells. One of the consequences is the previously mentioned Warburg effect, that promotes lactic acid accumulation, hyaluronic acid production and acidosis, while oxygen deprivation leads to hypoxia. These factors can contribute to the acquisition of resistance in tumor cells, by inducing expression of CD44 and suppressing immune activity within the niche (197). MYC profoundly drives metabolic reprogramming by enhancing glycolysis and stabilizing HIF1α, which leads to lactic acid accumulation and acidosis, suppressing cytotoxic T-cell function and polarizing macrophages toward immunosuppressive phenotypes in ovarian cancer models (198, 199). Studies indicate that combining immune checkpoint inhibitors with hypoxia mitigation enhances treatment efficacy and clinical outcomes (200). Metabolic reprogramming in tumor cells also influences cholesterol oxidation, producing metabolites such as hydroxycholesterol and epoxycholesterol that inhibit dendritic and effector T cells while promoting immunosuppressive populations. Adenosine, which is also secreted by tumor cells, can attach to A2A receptors on cytotoxic T cells, leading to their inhibition and enhancing resistance to immunotherapies (201, 202). In this context, oncogenic MYC also dysregulates cholesterol metabolism, increasing intracellular cholesterol and oxysterol production that impair dendritic and effector T-cell activity (203). Collectively, these metabolic alterations create a hypoxic, nutrient-deprived niche that reinforces immune exclusion and resistance to checkpoint blockade therapies (204).

During adaptive resistance, the activation of the immune response can be compensated by the polarization of tumor-associated macrophages (TAMs) and Tregs, both of which suppress effector T-cell activity. TAMs, particularly the M2 phenotype, secrete proangiogenic and immunosuppressive factors such as VEGF, IL-10 and TGF-β, potentiating immune evasion (205). As already mentioned before, MYC orchestrates M2 polarization by inducing secretion of these molecules (118–121, 206). Immunosuppressive Tregs inhibit cytotoxic T-cell proliferation through checkpoint expression and cytokine secretion (207). IL-10 suppresses antigen presentation and effector T-cell cytokine production, while TGF-β inhibits T-cell infiltration and promotes Treg differentiation (208, 209). Deregulated MYC also supports Treg proliferation and activation, inducing suppression of cytotoxic T-cell responses (210). In addition, these immunosuppressive chemokines recruit MDSCs, which impair T-cell responses through arginase and nitric oxide production. Tumors secrete chemokines such as CXCL5, CXCL8 and CCL2, engaging receptors CXCR2 and CCR2 on MDSCs, promoting their influx into tumors. As summarized in **Figure 3**, MYC orchestrates the secretion of these immunosuppressive cytokines and chemokines that attract MDSCs, amplifying immune suppression in acute myeloid leukemia cells (117, 211). This chemokine-driven recruitment constitutes a hallmark of adaptive resistance by dampening cytotoxic pathways while preserving tumor cell survival (212).

#### Acquired resistance

As mentioned before, acquired resistance is typically driven by epigenetic remodeling, EMT transition, stemness induction and mitochondrial or caspase alterations. Epigenetic silencing through DNA methylation often represses key pro-apoptotic genes, including BAX, PUMA and CASP8, impairing immune mediated cell death. This effect stabilizes survival signals and reduces tumor immunogenicity even after initial therapy success. Aberrant methylation reshapes apoptotic thresholds, promoting immune evasion and therapeutic relapse (213, 214). MYC regulates some of the pro-apoptotic mediators such as BAX, modulating apoptotic thresholds and facilitating survival under immune pressure (215). EMT enhances tumor plasticity, invasiveness and immune evasion. Induction of EMT-associated transcription factors, such as SNAI1/2, ZEB1/2 or TWIST, correlates with reduced antigen presentation and resistance to CD8+ T-cell killing (216). Concurrent stemness traits such as ALDH1A1, CD44 and MYC-driven PD-L1 expression promote recurrence and persistence following checkpoint therapy. This EMT-stemness axis fosters immune “cold” phenotypes and facilitates long-term acquired resistance (46, 47). Deregulated MYC can promote EMT and stemness by inducing transcription factors and stem cell markers, thus enhancing tumor plasticity in TNBC cellular models (217). Moreover, defective mitochondrial dynamics disrupt apoptotic signaling and metabolic integrity. Aberrant mitochondrial fission-fusion balance leads to a truncated mitochondrial outer membrane permeabilization (MOMP), producing sub-lethal caspase activation that triggers genomic instability and immune escape (218). MYC also controls mitochondrial structure and fission-fusion dynamics, influencing apoptotic sensitivity and genomic stability (219).

Regarding extrinsic mechanisms driving acquired resistance, chronic stimulation of immune checkpoints is a key factor. Prolonged exposure to ICI, such as anti-PD-1/PD-L1 or anti-CTLA-4, induces compensatory upregulation of secondary checkpoints, including TIM-3, LAG-3, TIGIT, BTLA and VISTA within tumor-infiltrating lymphocytes (220). Their overexpression marks terminal T-cell exhaustion, characterized by diminished cytokine release, impaired cytotoxicity and defective proliferation. Studies in melanoma and NSCLC show that elevated TIM-3 or LAG-3 expression in post-treatment samples correlates with relapse after PD-1 therapy (221). As illustrated in **Figure 3**, there is work showing the interaction between MYC and expression of immune checkpoint LAG-3 in TNBC (222), leading to immunosuppression via T-cell exhaustion.

## MYC inhibition to overcome IO resistance

Given MYC’s central role in driving oncogenic transcriptional programs and orchestrating immune evasion, therapeutic inhibition of MYC represents a promising strategy to restore tumor immunogenicity and sensitize tumors to immunotherapies. Nonetheless, efforts to directly target MYC have been hindered by its long-standing reputation as an “undruggable” protein, driven by multiple intrinsic features that challenge conventional drug discovery approaches. First, MYC is a nuclear transcription factor localized in the nuclei of cells, a compartment typically inaccessible to most compounds. Second, as a monomeric intrinsically disordered protein, MYC lacks well-defined hydrophobic pockets or enzymatic activity, precluding standard small molecule inhibitor design. Third, the MYC family comprises three paralogs (MYC, MYCN, and MYCL) that can partially compensate for one another, necessitating simultaneous inhibition for full therapeutic efficacy. Finally, MYC’s essential physiological role in normal cell growth and tissue regeneration has raised concerns that systemic inhibition could cause toxicity in healthy proliferative tissues. Despite these challenges, both direct and indirect strategies to modulate MYC activity have emerged, demonstrating promising preclinical and early clinical results.

### Indirect strategies

Indirect MYC inhibitors exploit vulnerabilities in MYC transcription, translation, or stability. There are clear advantages to these strategies, as some of the compounds used were originally developed for other oncogenic targets and are already available or clinically advanced. However, they may not be ideal for specific MYC inhibition due to limited selectivity and potential off-target effects. These indirect approaches are numerous and mechanistically diverse, and while they have been recently reviewed elsewhere (96, 100, 223–225), several stand out as particularly relevant in the context of immunotherapy resistance and are discussed below. Compounds with ongoing clinical evaluation are summarized in **Table 2**, while those with either completed, inactive, or discontinued trials are described in the text for completeness.

**Table 2:** Indirect MYC inhibitors under clinical development (only those with ongoing clinical trials are shown).

Class	Inhibitor	Mechanism of action (MoA)	Restoration of IO response	Developmental stage/clinical trials
G-quadruplex stabilizer	CX-5461 (Pidnarulex)	Stabilizes MYC promoter G-quadruplex, inhibits RNA Pol I, induces DNA damage and replication stress (226, 230)	Downregulates MYC transcription, increases tumor immunogenicity, enhances type I interferon signaling, boosts immune checkpoint blockade response (228, 229)	Phase I/II (solid tumors NCT06606990, NCT07147231, NCT07137416; hematologic malignancies NCT07069699); FDA Fast Track
CDK7 inhibitors	CT7001 (Samuraciclib)	Inhibit CDK7, block RNA Pol II phosphorylation and transcriptional initiation, suppress MYC transcription (241, 242)	Downregulate MYC and PD-L1, induce cell cycle arrest and apoptosis, sensitize tumors to anti-PD-1 therapy, enhance immune cell infiltration (245, 246)	Phase I/II (breast cancer NCT04802759; active not recruiting NCT06125522 and NCT05963997)
	SY-5609			Phase I/Ib (metastatic CRC NCT04929223)
CDK9 inhibitors	CYC065 (Fadraciclib)	Inhibit CDK9, block transcriptional elongation of MYC and anti-apoptotic genes, suppress MYC-driven oncogenic programs (244)	Downregulate PD-L1, induce apoptosis, increase immune cells in the tumor (248), potential synergy with IO therapies (247)	Phase I/II (Solid tumors and lymphoma NCT04983810, pediatric cancer NCT02813135)
	SCH 727965 (Dinaciclib)			Phase I (melanoma NCT00937937, solid tumors NCT01434316)
	BAY 1251152 (Enitociclib)			Phase I/II (lymphoid malignancies NCT05371054)
Aurora kinase A inhibitors	MLN8237 (Alisertib)	Inhibits Aurora A kinase, destabilizes MYC protein, disrupts mitosis and cell cycle progression (249–251, 255, 256)	Induce MDSCs and macrophage apoptosis, increase T cells, synergy with PD-L1 and CTLA-4 blockade (258, 259)	Phase II (breast cancer NCT06369285 and NCT02860000, SCLC NCT06095505, EGFR-mutant lung cancer NCT04085315). Phase III was stopped for futility (PFS endpoint).
	TAS – 119 (VIC-1911)			Phase I/II (hepatocellular carcinoma NCT06519721 and NCT05718882)
PLK1 inhibitors	Onvansertib	Inhibits PLK1, disrupts mitotic progression, indirectly destabilizes MYC (257)	Induces mitotic arrest and apoptosis, may enhance immune-mediated clearance of tumor cells (260–262)	Phase I/II (PDAC NCT06736717 and NCT04005690, CRC NCT06106308, leukemia NCT05549661, SCLC NCT05450965, breast cancer NCT05383196)
	CYC140			Phase I/II (solid tumors and lymphoma NCT05358379)
PP2A activators	LB-100	Restore tumor suppressor activity, target MYC for degradation (265) (266)	Downregulate MYC, restore immune surveillance, induce expression of neoantigens and enhance response to IO therapies (267, 269)	Phase I/II (ovarian cancer NCT06065462, CRC NCT06012734, sarcoma NCT05809830, SCLC NCT04560972, myelodysplastic syndromes NCT03886662)
BET inhibitors	AZD5153	Block BRD4-mediated chromatin regulation, suppress MYC transcription (279, 280, 291)	Increase tumor immunogenicity, restore interferon signaling, enhance antigen presentation, improve immunotherapy efficacy (281–286)	Phase I/II (acute myeloid leukemia NCT03013998)
	ZEN-3694			Phase I/II (solid tumors NCT06922318, NCT06102902, NCT05950464, NCT05803382, NCT05607108, NCT05422794, NCT05372640, NCT05327010, NCT05111561, NCT05071937, NCT05053971, NCT05019716, NCT04986423, NCT04840589)

G-quadruplex stabilizers such as CX-5461 (226) and CX-3543 (227) repress MYC transcription by stabilizing secondary DNA structures within the MYC promoter and ribosomal DNA, leading to replication stress, type I interferon activation, and increased tumor immunogenicity (228). Moreover, CX-5461 combined with anti-PD-1 or anti-PD-L1 enhanced therapeutic efficacy in CRC preclinical models (228, 229). CX-5461 has received FDA Fast Track designation and is being evaluated in multiple completed and ongoing phase I/II clinical trials, underscoring its therapeutic promise (230). Nonetheless, both agents suffer from off-target effects and unfavorable pharmacokinetics, with CX-3543 failing in clinical trials owing to limited bioavailability and strong plasma protein binding (227). Similarly, clinical trials with APTO-253, another G4-stabilizing agent, were halted due to manufacturing and solubility issues that led to a prolonged clinical hold, and ultimately the program was terminated due to insufficient clinical activity in the Phase I clinical trial (231). Others like IZCZ-3, QN-1, and IZTZ-1showed anticancer activity in preclinical models (232).

Similarly, CDK7 inhibitors [e.g. CT7001 (233), SY-1365 (234), SY-5609 (235), LY3405105 (236)) and CDK9 inhibitors (e.g. KB-0742 (237), CYC065 (238), dinaciclib (239), enitociclib (240)] suppress MYC transcriptional activity by blocking transcriptional initiation or elongation (241–244). Because inhibition of these transcriptional CDKs preferentially affects short-lived transcripts, including those encoding anti-apoptotic and cell cycle regulatory proteins, targeting this kinase family offers a rational strategy to blunt MYC-dependent transcriptional amplification (244). In addition to their direct antiproliferative effects, CDK7/9 blockade has been shown to downregulate PD-L1 expression, induce genomic instability triggering type I interferon responses, and enhance tumor immune infiltration, thereby sensitizing MYC-driven tumors to immune checkpoint blockade (245–248). For example, in SCLC, treatment with the CDK7i YKL-5–124 elicited immune response signaling and promoted activation of anti-tumor T cells and co-administration of YKL-5–124 with anti–PD-1 therapy led to a significant increase in overall survival compared with monotherapy (246). Similarly, in hepatocellular carcinoma (HCC), mice receiving the combination of a CDK9 inhibitor and an anti-PD-L1 antibody developed significantly smaller tumors and showed prolonged survival compared with those treated with either agent alone (247). Although preliminary pharmacodynamic data showing effective reduction of MYC expression are encouraging, as regulators of transcription, CDK7/9 inhibitors carry the risk of broad off-target effects by interfering with global transcription, which may constrain their therapeutic window. Ongoing clinical trials will be key to determine whether these inhibitors can selectively target MYC-driven cancers while maintaining an acceptable safety profile.

Other kinase inhibitors such as Aurora kinase A (Alisertib (249), MK-5108 (250), TAS-119 (251)) and PLK1 inhibitors (onvansertib (252), volasertib (253), CYC140 (254)) destabilize MYC at the post-translational level, triggering mitotic arrest and apoptosis (249, 250, 255), possibly enhancing immune recognition. Mechanistically, Aurora A kinase interacts with and stabilizes MYC by preventing its ubiquitin-mediated degradation; thus, Aurora A inhibition promotes MYC destabilization and proteasomal turnover (255, 256). Similarly, PLK1 inhibition interferes with MYC phosphorylation events required for its stability and transcriptional activity (257). By promoting MYC degradation, these compounds attenuate MYC-driven transcriptional programs and restore anti-tumor immune responses when combined with immunotherapies. For instance, alisertib has been shown to remodel the tumor immune microenvironment by depleting tumor-promoting myeloid populations and enriching cytotoxic T lymphocytes (258, 259). Moreover, it showed synergistic therapeutic effects when combined with PD-L1 and CTLA-4 blockade in models of breast (258) and papillomavirus-driven cancers (259). In the same line, PLK1 inhibitors have also been shown to exert synergistic effects in combination with immunotherapy (260). *In vivo* studies demonstrated that combining a PLK1 inhibitor with PD-L1 blockade markedly reduced tumor growth compared to either agent alone in both lung (261) and pancreatic cancers (262).

Another indirect strategy to inhibit MYC involves PP2A activators (e.g., DT-061 (263), LB-100 (264)), which restore phosphatase activity lost in MYC-driven cancers. Activation of PP2A promotes dephosphorylation of MYC at Ser62, leading to its proteasomal degradation (265, 266). By reducing MYC levels in this way, PP2A activators can restore antitumor immune surveillance and enhance tumor sensitivity to immunotherapies (267). In CRC, LB-100 treatment caused exon skipping and increased alternative splicing, inducing neoantigen expression (268) and, in a glioblastoma model, enhanced antitumor efficacy of PD-1 blockade (269).

BET inhibitors (e.g., JQ1 (270), OTX015 (271), CPI-0610 (272), GSK525762 (273, 274), BMS-986158 (275), INCB057643 (276), AZD5153 (277), ZEN-3694 (278)) block BRD4-dependent chromatin remodeling and MYC transcription, thereby restoring interferon signaling and antigen presentation, and are under active investigation in combination with immunotherapy (270, 279, 280). JQ1 was the first-in-class compound to establish proof-of-concept for BET inhibition in cancer therapy; however, its suboptimal *in vivo* pharmacokinetic profile has prevented its advancement to clinical trials. Despite this, preclinical studies have shown that JQ1 enhances antitumor immunity by upregulating MHC-I expression, downregulating PD-L1, and synergizing with anti-PD-1/L1 therapies across multiple cancer models (281–286). Despite the promising preclinical activity of BET inhibitors, clinical translation has proven challenging. The majority of compounds have been discontinued or had their clinical trials terminated due to limited efficacy or tolerability issues. Furthermore, BET inhibitors downregulate other genes beyond MYC, suggesting that their anticancer effects are not solely MYC-dependent (66). Only a few agents, such as AZD5153 and ZEN-3694, remain under active clinical investigation. Notably, in preclinical models, AZD5153 has been shown to promote antitumor immunity by depolarizing immunosuppressive M2 macrophages (287) and increasing sensitivity to anti-PD-L1 therapy (288).

Finally, epigenomic modulation has also been pursued to indirectly target MYC. OTX-2002 is a first-in-class mRNA-based epigenomic modulator that represses MYC pre-transcriptionally by establishing targeted epigenetic marks. Preclinical studies in HCC demonstrated strong anti-tumor activity, downregulation of PD-L1 and improved therapeutic activity when combined with anti-PD-(L)1 antibodies. Interestingly, the therapeutic effect was accompanied by a reduction of Tregs in the TME (289). Preliminary clinical data indicate a favorable safety profile and on-target epigenetic effects (290).

### Direct strategies

The challenges of indirect MYC inhibitors have driven the pursuit of direct MYC-targeting approaches designed to engage the oncoprotein itself, offering a more selective and mechanistically precise means of inhibiting MYC and restoring tumor immune surveillance. Direct MYC inhibitors aim to disrupt MYC function at the protein level, either by preventing its dimerization with MAX, blocking DNA binding, or promoting MYC degradation (100, 223–225). Such strategies once thought unfeasible have now entered preclinical and early clinical evaluation, showing promising signs of efficacy and tolerability. The following section highlights the most advanced and promising direct MYC inhibitors, with those currently undergoing clinical testing summarized in **Table 3**.

**Table 3:** Direct MYC inhibitors under clinical development (only those with ongoing clinical trials are shown).

Class	Inhibitor	Mechanism of action (MoA)	Restoration of IO response	Developmental stage/clinical trials
Mini-protein	OMO-103 (Omomyc)	Cell-penetrating mini-protein; disrupts MYC–MAX dimerization, blocks DNA binding (292, 293)	Increases T-cell infiltration, reprograms macrophages to M1 phenotype, decrease PD-L1 expression (295, 299, 300)	Phase Ib/II (PDAC NCT06059001, NCT07089940; high-grade osteosarcoma NCT06650514)
Stapled peptide	IDP-121	Disrupts MYC–MAX dimerization and induces c-MYC degradation (301, 302)	Unknown	Phase I/II (hematologic malignancies NCT05908409)
Small molecule degrader	WBC100	Targets c-MYC for proteasomal degradation by binding to its NLS1–Basic–NLS2 region (307)	Unknown	Phase I (acute myeloid leukemia NCT07014449, solid tumors NCT05100251)

The most advanced direct MYC inhibitor in clinical trials is OMO-103, a MYC dominant negative. It is the clinical formulated drug product derived from Omomyc, a 91 amino acid mini-protein based on the MYC b-HLH-LZ domain engineered with four amino acid substitutions that alter its dimerization properties (292). Omomyc can form both homodimers and heterodimers with MYC and MAX. While Omomyc/Omomyc and Omomyc/MAX dimers bind E-box sequences as transcriptionally inactive complexes, Omomyc/MYC dimers cannot bind DNA (292, 293). Through this dual mechanism of action - sequestering MYC away from DNA and occupying its target promoters with repressive complexes - Omomyc acts as a dominant-negative inhibitor of MYC transcriptional activity. Extensive preclinical studies have demonstrated that Omomyc-mediated MYC inhibition is both highly effective and well tolerated, suppressing tumor growth and inducing regression across multiple cancer types, irrespective of tissue origin or oncogenic driver (49, 52, 113, 294–298). In line with MYC’s capacity to drive tumorigenesis through both cell-intrinsic and -extrinsic mechanisms, Omomyc exerts dual anticancer actions directly suppressing tumor cell growth while simultaneously reshaping the surrounding microenvironment. For example, Omomyc treatment in a KRAS-driven NSCLC mouse model increased immune infiltration, notably recruiting T cells to the tumor site (295). Additionally, a phylomeric form of Omomyc reduced PD-L1 expression in a model of TNBC (299). Beyond cancer models, Omomyc-mediated c-MYC inhibition in mature macrophages triggered extensive transcriptomic remodeling in a tuberculosis model, upregulating key inflammatory pathways such as IFNγ, TNFα, and IFNα responses, and underscoring its broad immunomodulatory potential (300). Together, these effects contribute to a more immunostimulatory tumor milieu and may sensitize tumors to immune checkpoint blockade. OMO-103 is now being evaluated in Phase Ib/II clinical trials.

IDP-121 and IDP-410 are next-generation stapled peptide MYC inhibitors that target distinct members of the MYC oncogene family, c-MYC and N-MYC respectively. Both compounds disrupt MYC’s interaction with MAX and promote MYC monomer degradation. IDP-121 has demonstrated antitumor activity across multiple preclinical hematologic and solid tumor models (301) and has recently entered clinical evaluation, while IDP-410 reduced growth and vascularization *in vivo* in a glioblastoma mouse model, suggesting a link between N-MYC function and mesenchymal or angiogenic programs (302). To date, no further public data have been released for either compound, and their potential effects on the tumor immune microenvironment remain unexplored.

Other small molecules have been developed to target the MYC–MAX interaction, including MYCi361 and its optimized analogue MYCi975. These compounds disrupt MYC–MAX dimerization and promote phosphorylation of MYC at T58, leading to proteasome-mediated degradation (303). In preclinical models, MYCi361 suppressed tumor growth and enhanced immune infiltration, increasing CD3^+^ T cells, IFNγ^+^ CD4^+^ and CD8^+^ T cells, TNFα^+^ CD8^+^ cells, dendritic cells, and NK cells, while upregulating PD-L1 and sensitizing tumors to anti-PD-1 therapy. Although MYCi361 displayed a narrow therapeutic window, MYCi975 showed improved tolerability while retaining immunomodulatory activity, including increased pT58, PD-L1 expression, and enhanced infiltration of T, B, and NK cells. Combination therapy with anti-PD-1 yielded synergistic tumor suppression (303). Similarly, in TNBC and head-and-neck cancer mouse models, MYCi975 increased CD8^+^ T-cell infiltration (304, 305), and in TNBC, co-treatment with anti-PD-L1 produced stronger tumor growth inhibition than either agent alone (304). An optimized version of MYCi975 is expected to enter clinical evaluation in the near future (306).

Other direct MYC-targeting strategies aim to destabilize MYC by promoting its degradation. One such approach involves the small-molecule degrader WBC100, which targets MYC proteostasis rather than dimerization. However, for MYC, this strategy is challenging due to the protein’s inherently short half-life and continuous synthesis in cancer cells, which could necessitate frequent or sustained dosing of any therapy that relies solely on MYC degradation. In preclinical models, oral WBC100 administration markedly reduced MYC protein levels, inhibited tumor growth, and prolonged survival without notable toxicity (307). The compound is currently being evaluated in a Phase I clinical trial for patients with advanced solid tumors overexpressing c-MYC. To date, however, no clinical data or evidence regarding its potential effects on the tumor immune microenvironment have been reported.

Alternative strategies to inhibit MYC focus on blocking its mRNA translation using antisense oligonucleotides or RNA interference (RNAi) technologies such as siRNA or shRNA. Among these, DCR-MYC, a MYC-targeting siRNA, advanced to Phase I/II clinical trials but was discontinued due to insufficient gene-silencing efficacy (308). Although the immunological effects of MYC-targeted RNA therapies have not been directly characterized, MYC silencing is expected to indirectly enhance antitumor immunity by reversing MYC-driven immunosuppression. Thus, future studies integrating RNA-based MYC inhibition with immune checkpoint blockade could provide valuable insights into their potential immunomodulatory synergy.

While this review focuses on the immunomodulatory effects of MYC inhibition, it is important to note that many of the compounds discussed above also impact classical tumor-intrinsic mechanisms of oncogenesis induced by MYC. MYC-targeted therapies can suppress proliferation, induce apoptosis, and impair metabolic programs that are critical for tumor growth and survival. For example, CDK7/9 inhibitors, Aurora kinase A inhibitors, and direct MYC inhibitors such as Omomyc not only enhance tumor immunogenicity but also reduce MYC-driven transcriptional amplification of genes involved in cell cycle progression, ribosome biogenesis, and DNA replication. However, the extent to which each strategy affects the full spectrum of MYC functions varies, reflecting differences in mechanism of action and target specificity. Understanding how MYC inhibition balances tumor-intrinsic and immune-mediated effects will be crucial for choosing the most optimal combination therapies.

## Conclusions

MYC plays a critical role not only in tumor initiation, progression, and maintenance but also as a central driver of immune evasion and resistance to immunotherapies. By orchestrating multiple cell-intrinsic and microenvironmental tumorigenic programs, MYC enables cancer cells to evade immune surveillance and adapt to therapeutic pressures. Its deregulation disrupts antigen presentation, alters signaling pathways, remodels the tumor microenvironment, and promotes immunosuppressive metabolic and cellular conditions that collectively undermine anti-tumor immunity. The broad influence of MYC across these diverse mechanisms highlights its critical position as a master regulator of resistance to immune checkpoint blockade and other immunotherapies.

Notably, MYC is rarely the primary driver mutation in cancer, and immune suppression often emerges in the context of co-occurring oncogenic events and tumor suppressor alterations. In this setting, MYC frequently acts downstream or in parallel to initiating oncogenic lesions, integrating signals from co-mutations and tissue-specific cues to shape both tumor-intrinsic programs and the tumor immune microenvironment. Consequently, the relationship between MYC activation and immune dysfunction can reflect both direct MYC-driven effects and immune suppression that develops in parallel during transformation, in a manner that is highly tumor- and tissue-dependent.

Finally, MYC is currently being targeted both directly and indirectly through emerging therapeutic strategies, offering promising avenues to overcome immunotherapeutic resistance. Direct inhibitors of MYC and agents targeting its regulatory pathways have demonstrated the ability to restore immune sensitivity and enhance immunotherapy efficacy in preclinical models. These findings underscore the potential of MYC inhibition as a powerful adjunct to current immunotherapies, aiming to improve patient outcomes by reversing immune escape and resistance mechanisms in cancer.
